# Beneficially Stressing the Peripheral Nervous System to Repair

**DOI:** 10.3390/ijms27146327

**Published:** 2026-07-16

**Authors:** Valerie M. K. Verge, Zhengxin Ying, Wafa A. Mustafa, Justin M. Naniong, Joelle R. Nadeau, Jovan C. D. Hasmatali, Miles E. Magno, Vikram Misra, Gillian D. Muir

**Affiliations:** 1 Department of Anatomy, Physiology and Pharmacology, University of Saskatchewan, Saskatoon, SK S7N 5E5, Canada; yingzhengxin@cau.edu.cn (Z.Y.); wam733@mail.usask.ca (W.A.M.); justin.naniong@mail.mcgill.ca (J.M.N.); primalcarecenter@gmail.com (J.R.N.); jhasmatali@manitoba-physicians.ca (J.C.D.H.); mem807@mail.usask.ca (M.E.M.); 2 Cameco MS Neuroscience Research Centre, University of Saskatchewan, Saskatoon, SK S7K 0M7, Canada; gillian.muir@usask.ca; 3 Department of Veterinary Microbiology, WCVM, University of Saskatchewan, Saskatoon, SK S7N 5B4, Canada; vikram.misra@usask.ca; 4 Department of Veterinary Biomedical Sciences, WCVM, University of Saskatchewan, Saskatoon, SK S7N 5B4, Canada

**Keywords:** sensory neuron, regeneration, electrical nerve stimulation, acute intermittent hypoxia, myelination, endoplasmic reticulum stress, unfolded protein response, Luman, CREB3, motor neuron

## Abstract

Peripheral neurons have an intrinsic capacity for repair, albeit still challenging. How the nervous system responds to the cellular stress imposed by nerve injury and adjunct therapies impacts axon regeneration and functional outcomes. Here, we summarize some of the key primarily axonal and neuronal adaptive stress responses and mechanisms that underlie the ability of peripheral neurons to regenerate an axon. This includes activation of the unfolded protein response and endoplasmic reticulum membrane-associated molecules, namely Luman/CREB3, a transmembrane basic leucine zipper transcription factor that regulates the encoding of beneficial adaptive stress responses that drive the ability of an injured sensory neuron to regenerate their axon. We also highlight an emerging novel non-invasive strategy, therapeutic acute intermittent hypoxia, that imposes a level of beneficial adaptive stress that can alter gene programs induced by the injury to enhance regeneration in a manner akin to the more invasive yet highly effective electrical nerve stimulation. The ability to manipulate and significantly elevate the intrinsic adaptive stress/repair responses of injured peripheral neurons holds therapeutic promise, with accumulating evidence supporting its clinical use.

## 1. Introduction

Despite over 200 years of research into peripheral nervous system (PNS) regeneration, there are still many challenges associated with recovering from a nerve injury [1,2].

The act of injuring a peripheral nerve axon imposes a level of cellular stress that is necessary to transition the neuron from a homeostatic state to a regenerating state. As our understanding of PNS regeneration and its inherent challenges, such as loss of function and neuropathic pain states, become better elucidated, along with the development of therapies that enhance the intrinsic capacity for repair, we are arriving at a time of hope for patients with traumatic PNS injury.

This review focuses on the adaptive stress responses believed to underlie the ability of PNS neurons to regenerate and how the stress response from an injury is encoded at the somal and axonal endoplasmic reticulum (ER), as the neuron attempts to regenerate its axon and survive the insult. Researchers are beginning to understand how ER stress, namely the unfolded protein response (UPR), is critically involved in axon regeneration and how the signals are generated. We also explore a novel regulator of the response, Luman/CREB3, that is synthesized and cleaved at the site of injury and serves as a retrograde signal that transcriptionally regulates a beneficial adaptive UPR response critical for regenerating the axon in injured sensory neurons [3,4]. Finally, we will discuss a novel non-invasive therapy called therapeutic acute intermittent hypoxia (tAIH), which we find appears to induce a beneficial adaptive stress response, enhancing the intrinsic regeneration response in peripheral neurons in a manner akin to the current gold standard electrical nerve stimulation (ES) [5], the latter discussed in detail in Gordon, 2024 [6].

The marked increases in somal and axonal protein synthesis in response to nerve injury invokes a number of adaptive responses to ensure that the proteins produced are properly folded and functional as they leave the ER and get transported to their destinations [7]. Misfolded or unfolded proteins are either refolded or degraded. However, an accumulation of unfolded proteins in the ER can activate the unfolded protein response (UPR) as the neuron attempts to re-establish proteostasis and regenerate its axon.

## 2. The Unfolded Protein Response

In eukaryotes, proteins must be properly folded in the ER lumen before delivery to their destination. Axon injury results in greatly elevated levels of newly synthesized polypeptide entering the ER. This can create an imbalance between the folding capacity of the ER and the increasing numbers of proteins requiring this, creating ER stress. As correct protein folding is critical, understanding this ER stress pathway and its regulators gives us insights into how to optimally drive a beneficial adaptive response [8]. There are signal pathways that detect and adapt protein folding status and that collectively are referred to as the UPR (reviewed in [9]). It is important for cells to adapt to alterations in proteostasis induced by nerve injury, with strategies including slowing down overall protein synthesis, increasing the amount of ER and degrading misfolded proteins (reviewed in [10]). However, if cells fail to re-establish homeostasis, the UPR triggers apoptosis to protect cells from the dysfunction caused by unfolded protein accumulation.

There are three main transducers that initiate the UPR. While these form three distinct arms of the UPR, they are highly interactive and regulatory in their roles. The three main UPR transducers are inositol-requiring protein-1 (IRE1), protein kinase RNA-like ER kinase (PERK), and activating transcription factor 6 (ATF6).

### 2.1. IRE1

The first ER stress transducer described was IRE1, a type 1 ER-resident transmembrane protein that is conserved even in lower eukaryotes [11,12]. IRE1 functional domains include a cytoplasmic kinase module, an endoribonuclease (RNase) domain, and an ER luminal domain that associates with the chaperone protein, glucose-regulated protein 78 (GRP78; aka BiP). Whether IRE1 activates depends on the dissociation of GRP78 that occurs when there is an increased presence of unfolded proteins competing for this interaction; it is not a direct interaction of IRE1 with the unfolded proteins [13,14]. The dissociation of GRP78/BiP allows IRE1 oligomerization and transphosphorylation of its kinase domain [15]. Instead of generating a sequential kinase cascade [16], IRE1 phosphorylation triggers its endoribonuclease activity, resulting in the unconventional splicing of X-box binding protein 1 (XBP1) mRNA [17], the endonucleolytic decay of select mRNAs [18,19], and a reduction in the flow of newly synthesized peptide chains into ER with the intent of hopefully adapting to the ER stress.

The resulting frameshift from the splicing of XBP1 mRNA [20] leads to a protein with different functions. The translation of XBP1splice (XBP1s) increases with IRE1 activation [17]. The contrary applies to the unspliced form of XBP1 (XBP1u), whose translation is enhanced with IRE1 inactivation. The resulting XBP1u protein inhibits the UPR by complexing with XBP1s, leading to the rapid degradation of the complex [20]. In this manner, XBP1u negatively regulates XBP1s, terminating its transcriptional role as the cell recovers from ER stress [20].

XBP1s belongs to the basic leucine zipper (bZIP) transcription factor family, binding to the ER stress response element (ERSE) [21] and UPR element (UPRE) [22] in target genes. XBP1s regulates several processes in the ER stress response, including the generation of phospholipids, biogenesis of additional ER to meet the increasing protein folding demand [23], and elevation of UPR-related gene expression [24]. Signal recognition particle 54 (SRP54) is one of these target genes that assists the ER entry of polypeptides [25], while others such as ERdj4, another chaperone protein and ER degradation-enhancing alpha-mannosidase (EDEM) participate in the ER-associated degradation (ERAD) response aimed at ridding the cell of misfolded protein [26], while the XBP1s target protein, disulfide isomerase, serves to catalyze protein folding [27].

### 2.2. PERK

Like IRE1, PERK is classified as a type I ER transmembrane protein. Its structural domains include a stress-sensing ER luminal domain that subserves a similar function to that of IRE1 [28], and protein kinase activity on the cytoplasmic side [29]. It also is held inactivated by binding GRP78/BiP on its luminal side. Its activation results from a release of GRP78/BiP due to an increased production of unfolded proteins requiring GRP78/BiP as a chaperone. When GRP78/BiP is not associated with PERK, PERK oligomerizes and transphosphorylates, activating its kinase domain, which then targets and phosphorylates the eukaryotic translation factor 2 (eIF2), dampening its ability to initiate translation [29]. This reduction in general protein translation reduces the level of ER stress, helping the cell adapt and regain proteostasis.

However, not all protein synthesis is stopped when eIF2 is phosphorylated (peIF2). ATF4 escapes this control as its mRNA contains open reading frames in its 5’ region that permit its translation in the presence of peIF2 [30]. As an activated transcription factor ATF4, it binds CCAAT-enhancer binding protein-activating transcription factor response elements (CARE) targeting CCAAT-enhancer-binding protein homologous protein (CHOP) [31] and DNA damage-inducible protein 34 (GADD34) [32] transcription.

When PERK signaling is prolonged and CHOP levels become high, CHOP in its role as a transcription factor promotes the expression of pro-apoptotic genes (reviewed in [33]) that lead to the loss of the cell rather than having one that is dysfunctional due to the presence of many misfolded or unfolded proteins. Another option is to express higher levels of GADD34, which by binding and activating type I protein phosphatase (PP1) [34] effects eIF2 dephosphorylation [35], serving as a negative regulator of the PERK response and allowing protein translation.

### 2.3. ATF6

The ATF6 arm acts as an ER stress transducer in a different manner. As a type II ER transmembrane protein, ATF6’s luminal domain is also regulated by a competition for GRP78/BiP binding with the unfolded proteins being generated [36]. Unlike IRE1 and PERK, its cytoplasmic domain contains a bZIP motif, allowing it to also act as a transcription factor when this domain is cleaved and released [37]. The dissociation from GRP78/BiP when under ER stress unmasks Golgi localization signals that translocate it to the Golgi apparatus [38] via coat protein II (COPII) transport vesicles [39], a response influenced by ATF6 glycosylation [40] and disulfide bonds states [41]. Once in the Golgi, the cleavage of ATF6 is by site-1 protease (S1P) and site-2 protease (S2P). This releases its N-terminal cytoplasmic domain [42], allowing this domain, a bZIP transcription factor, to travel to the nucleus and effect the transcription of target genes containing ER stress elements (ERSEs) [43]. These include proteins involved in the ER stress response such as GRP78/BiP (a molecular chaperone), Derlin-3 (an ER-associated degradation system-associated protein) [44], CHOP and XBP1 [43]. In addition, ATF6 regulates the expression of phospholipids and the generation of more ER [45,46].

A growing number of proteins with ATF6-like structures that also localize to the ER are being identified. These also serve as bZIP transcription factors and include Luman/CREB3 [47], OASIS/CREB3L1 [48], CREB3L2 [49], CREB3L4 [50] and CREBH [51]. These proteins also appear to subserve tissue- and response element-selective roles in regulating and finetuning UPR responses [reviewed in [52,53]].

## 3. The UPR and Cholesterol-Important Roles in Repair and ER Stress

The UPR also subserves a vital role in maintaining lipid homeostasis associated with the need to generate more ER membrane in response to ER stress to cope with the increased protein folding demand. But when one considers what is required for cell specific events such as axon regeneration, it must also regulate the associated increased protein and lipid production. This is a tightly regulated response that is activated by phospholipid depletion [54] and membrane lipid saturation [55]. ER stress also up-regulates lipid biogenesis by controlling rate-limiting enzymes involved in this process [56], supporting a role for the UPR in lipid metabolism. ER membrane expansion requires lipid biosynthesis and helps in adapting to the stress imposed by unfolded protein accumulation [57]. XBP1 appears linked to this response as increasing XBP1 expression leads to elevated levels of membrane phospholipids and ER expansion [23]. Interestingly, the UPR also senses whether sterol levels are sufficient by controlling the expression and activation of ER membrane resident transcription factors called sterol regulatory element-binding proteins (SREBPs) (reviewed in [58]) [42].

### 3.1. SREBP and Cholesterol Regulation

Cholesterol is critical for cell membrane function, subserving important roles in membrane fluidity and permeability. Its levels are tightly regulated in cells, as at high levels it is toxic [reviewed in [59]]. How exogenous cholesterol enters the cell is also regulated, involving an interaction between low-density lipoprotein (LDL) and the LDL receptor. Once bound to its receptor, LDL is internalized into cells. Cellular synthesis of cholesterol is a means to increase endogenous levels, requiring several SREBP-regulated enzymes [60].

SREBP activation involves cholesterol association with the SREBP cleavage-activating protein (SCAP) [61], creating a complex that is localized to the ER membrane via a SCAP–protein insulin-induced gene (INSIG) interaction, which dissociates when cholesterol levels decrease [62,63]. This allows SREBP/SCAP complex translocation to the Golgi apparatus [64] in a COPII-vesicle-dependent manner [65]. Like ATF6 and other ER-resident bZIP transcription factors, SREBP is processed in the Golgi by S1P and S2P [60,61,66], releasing the N-terminal cytoplasmic transcription factor domain that regulates target genes with the sterol regulatory element. These include the LDL receptor [67] and HMG-CoA reductase [60], the cholesterol biosynthetic rate-controlling enzyme. Part of the cholesterol metabolism negative feedback loop involves SREBP regulation of proprotein convertase subtilisin/kexin type 9 (PCSK9) expression [68], leading to increased LDL receptor internalization and degradation [69].

### 3.2. UPR-Associated SREBP Activation

The shared activation of SREBP and ATF6 by regulated intramembrane proteolysis (RIP), suggests common regulatory processes, with SREBP activation and cholesterol accumulation increasing in response to a heightened UPR/ER stress [70]. Increased cholesterol levels can also activate the UPR and regulate apoptotic pathways by increasing CHOP expression in an ATF4-driven response [71]. There is also a link between the UPR and SREBP activity. The suppression of general protein synthesis by eIF2 phosphorylation results in reduced INSIG expression [72], allowing an SREBP release from the ER membrane, leading to its activation [72]. In contrast, elevated GRP78/BiP levels mitigate UPR SREBP activation [70] by maintaining the interaction between these two proteins [73]. So, like ATF6, the interaction of SREBPs with GRP78/BiP in addition to INSIG prevents their translocation to the Golgi and subsequent activation.

Additional mechanisms involved in the adaptation to increasing ER stress are: (i) to invoke translocation of the proteins to the cytosol where they can be tagged with ubiquitin and degraded in proteosomes as part of the ER-associated degradation (ERAD) response [74]; or to invoke the complementary response of (ii) selective autophagy, whereby large protein aggregates or portions of the ER are delivered to lysosomes for protein degradation or recycling [75]. Autophagy has been described as comprising three forms—microautophagy, macroautophagy and chaperone-mediated autophagy—with the most studied being macroautophagy [76,77] and herein called autophagy.

Emerging evidence reveals that the ability of injured neurons to adapt to the ER stress imposed by peripheral nerve injury, regain proteostasis, and rid the cell of misfolded and damaged proteins and organelles through ERAD and autophagy are all necessary steps in the regenerative response, as discussed below.

## 4. UPR Activation and Nerve Regeneration

Activation of the UPR is now observed in many neurodegenerative disorders, such as Alzheimer’s disease [78,79], Parkinson’s disease [80], and multiple sclerosis [81]. The manipulation of select UPR components such as CHOP deletion or XBP1 activation [82,83] have also been linked to retinal ganglion cell survival, the latter also promoting the survival of Schwann cells in injured nerves [84].

**Figure 1:**
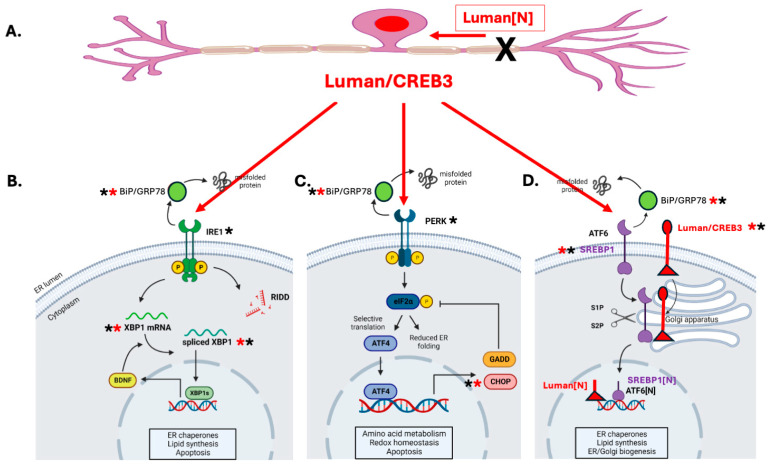
**Axonal Luman/CREB3 injury-induced expression and activation serves as a retrograde signal that regulates a beneficial adaptive UPR critical for axon regeneration.** (**A**) Sensory neuron peripheral axon injury (**X**) induces axonal Luman/CREB3 transcript translation and activates ER-membrane-localized Luman/CREB3 to release the N-terminal of Luman (Luman[N]), a transcription factor that serves as an important retrograde signal traveling back to the cell soma and nucleus (red) where it regulates axon regeneration and a beneficial adaptive response in the 3 arms of the UPR ((**B**–**D**); red arrows) linked to nerve regeneration [3,4,96]. Note: UPR proteins shown to be regulated by nerve injury are denoted by a black asterisk (*), while those shown to be regulated by injury and Luman/CREB3 are identified with a red asterisk (*) (**B**) *IRE1 arm of the UPR system*. The IRE1 arm is activated by oligomerization and phosphorylation of IRE1 due to BiP/GRP78 dissociation and binding to increased levels of misfolded protein in the ER. Activated IRE1 initiates unconventional XBP1 mRNA splicing and RIDD activity to decrease ER protein load. Spliced XBP1 functions as a transcription factor to initiate genes important for ER chaperone synthesis, lipid synthesis, and, in cases of sustained ER stress, apoptosis. (**C**) *PERK arm of the UPR system*. Activation of the PERK arm initiates when Bip/GRP78 dissociates from PERK due to association with unfolded proteins allowing its oligomerization and phosphorylation. Activated PERK initiates phosphorylation of eIF2α, which slows general translation to reduce protein load, while promoting production of the transcription factor ATF4. ATF4 activates genes associated with amino acid metabolism, redox homeostasis, and, in cases of sustained ER stress, apoptosis via CHOP. GADD transcription products dephosphorylate eIF2α. (**D**) *ATF6 arm of the UPR*. The ATF6 arm is initiated by the translocation of the ATF6 protein to the Golgi due to BiP/GRP78 dissociation and binding to increased levels of misfolded protein in the ER. In the Golgi, ATF6 gets cleaved in two distinct regions by S1P and S2P proteases. The ATF6 N-terminal (ATF6[N]) translocates into the nucleus to initiate gene transcription and activate pathways involved in ER chaperone production, lipid synthesis, and ER/Golgi biogenesis. Luman/CREB3 and SREBP1 are also ER-membrane-localized transcription factors that, with ER stress, activate by translocating to the Golgi and have their N-terminal transcription factor domains released by RIP (Luman[N] and SREBP1[N]) similar to ATF-6, serving as regulators of the UPR and cholesterol biosynthesis. BiP = binding immunoglobulin protein (aka GRP78); IRE1 = inositol-requiring enzyme 1; XBP1 = X-box binding protein 1; XBP1s = spliced X-box binding protein 1; BDNF = brain-derived neurotrophic factor; RIDD = regulated IRE1-dependent decay/degradation. PERK = protein kinase R-like ER kinase; eIF2α = eukaryotic initiation factor 2 protein; ATF4 = activating transcription factor 4; CHOP = C/EBP homologous protein; GADD = growth arrest and DNA damage protein; ATF6 = activating transcription factor 6; S1P = site-1 protease; S2p = site-2 protease; ATF6[N] = N-terminal domain of ATF6 protein; SREBP1 = sterol regulatory element binding protein-1. Image created using BioRender.

Despite a capacity for regeneration, injured peripheral nerve axons are still fraught with challenges that impact functional recovery, including injury severity, delayed repair, and age of the patient [85,86]. Complex cellular responses, including calcium influx [87], histone acetylation [88], axonal protein synthesis [89], retrograde [89] and anterograde [90] axonal transport of injury signal mRNAs and newly synthesized proteins [89,91], occur in response to nerve injury [92]. Emerging evidence supports the idea that UPR activation in axons is possible. This includes an ability for brain-derived neurotrophic factor (BDNF) application to axons to induce axonal XBP1 splicing, driving neurite outgrowth, which is significantly curtailed in XBP1−/− mice [93], and lysophosphatidic acid treatment resulting in axonal eIF2 phosphorylation [94]. The UPR is also activated in dorsal root ganglion (DRG) neurons and axons one day after sciatic nerve crush injury, at both transcript and protein levels [4] (Figure 1). More recently, the injury response has been shown to drive an accelerated axonal ER calcium release, with inhibition of this release impairing regeneration [95]. Roles for individual components of the UPR have also been implicated in nerve regeneration events. The IRE1/XBP1 axis has been linked to nerve regeneration, functional recovery, and myelin clearance and macrophage infiltration in the Wallerian degenerating nerve [96]. Selective inhibition of the PERK and IRE1 pathways also indicate roles in ER and growth cone morphology and dynamics, including axonal ER fragmentation [95]. As well, neuronal overexpression of the UPR target gene Erp57, a disulfide isomerase that can function as both a chaperone and a glycoprotein foldase, improves axon repair following sciatic nerve crush by promoting an enhanced immune response, axon regeneration, myelin clearance and functional recovery [27].

Other important cellular processes involved in adapting to cellular stresses are ERAD and autophagy. Indeed, mutations in genes involved in autophagy are implicated in a growing number of neurodegenerative diseases [97,98]. Roles for ERAD and autophagy are also emerging as important and necessary processes in peripheral nerve regeneration. These include the clearing of axonal and myelin debris in the Wallerian degenerating portion of the nerve distal to injury [77,99], the promotion of myelin homeostasis and maintenance [100], and the mitigating of neuropathic pain states [101]. When a nerve is injured, the Schwann cells distal to the injury dedifferentiate, express the molecule, mixed lineage kinase domain-like protein (MLKL) that initiates myelin breakdown [102] and begin clearing myelin debris in an autophagic process referred to as myelinophagy, driven by the JNK/c-jun pathway activated in these cells [103]. This, combined with the generation of chemokine signals that recruit monocytes to the area that differentiate into phagocytic macrophages, helps efficiently clear the region of myelin and axonal debris to promote axon regeneration [103,104].

Elucidating the cellular processes underlying the UPR gives insight into how injured neurons adapt to the task of having to regenerate axons following nerve injury. This, coupled with our discovery that ER-resident Luman/CREB3, an ATF6-like protein, is critically involved in axon regeneration [3], led to investigations into whether there might be links between the UPR and Luman/CREB3 in nervous system repair.

## 5. Luman/CREB3—Structure and Regulation

Initial investigations of Luman (aka CREB3 or LZIP, herein referred to as Luman) revealed it to interact with the host cell factor (HCF), a scaffold protein that regulates herpes simplex virus infection [47,105]. Like ATF6, Luman is an ER transmembrane protein, belonging to the bZIP transcription factor superfamily and is activated in a similar manner by RIP once it translocates to the Golgi in response to stress. Known functions of Luman outside axon regeneration include the regulation of dendritic cell maturation and cell migration [106] and the UPR (see below).

The transcription domain activated by Luman contains three elements: two LxxLL motifs, found in many transcription factors, associated cofactors that facilitate protein–protein interactions, and a HCF-binding motif [107]. There is also a conserved sequence that is located adjacent to the N-terminal of the bZIP domain found in multiple other members of the CREB3 family, including OASIS/CREB3L1, CREB3L2, CREB3L4 and CREBH. However, this conserved sequence is not found in typical bZIP transcription factors, including the UPR-associated ATF6 (reviewed in [52]). Like ATF6, Luman translocates to the Golgi for activation, which requires cleavage and the release of the N-terminal by S1P and S2P (hereby designated Luman[N]) [108]. Notably, the paucity of homology of Luman’s ER luminal domain and that of ATF6 implies that the ways in which stress is sensed to ultimately activate each of these bZIP transcription factors differ. Unlike ATF6 and SREBP1, Luman does not appear to be held inactive through a GRP78/BiP interaction [109].

Luman has a molecular weight of ~60 kDa in its inactivated state in the ER membrane [110]. Activation releases the ~40 kDa N-terminal containing the transcriptional regulatory domains [108]. Genes regulated by Luman[N] contain cAMP response element (CRE), unfolded protein response element (UPRE) and ER stress element (ERSE) II in the promoter region. The degree of Luman-driven gene activation can be negatively regulated by the Luman recruitment factor [111,112] and Jun activation domain-binding protein 1 [113], which result in the rapid degradation of Luman and likely underlies why it is only transiently observed within the nuclear compartment.

That Luman plays a role in the adaptive response to ER stress is supported by its ability to protect against UPR-associated apoptosis when it is over-expressed [110] and also its ability to regulate the expression of two UPR ERAD-associated genes, homocysteine-induced endoplasmic reticulum protein (HERP) [110] and ER degradation-enhancing alpha-mannosidase (EDEM) [114]. Given that EDEM [26] and HERP [115] have roles in protein degradation [115,116], it supports a link between Luman and ERAD.

Prior to direct investigations into the role of Luman in nerve injury, there was little evidence that it played a significant role in the nervous system. It was known to be expressed by primary sensory neurons, where it was involved in the reactivation of the herpes simplex virus (HSV), initiated by a neuronal stress response [117]. We posited that Luman might serve as a sentinel, sensing and encoding stress responses to injury in the peripheral nervous system. Further, in a transcriptome analysis of adult rat sensory axons, Luman transcripts had been identified but remained to be validated [118], prompting us to create tools to assess its role in stress responses to peripheral nerve injury in both axonal and somal compartments.

Cloning of the entire rat Luman coding sequence [119] revealed it to be 387 amino acids long, 233 amino acid residues longer than that reported previously (GenBank: BC062241.1), aligning 85% with mouse and 66% with human sequences. Importantly, it revealed all of the previously identified functional domains to be conserved, supporting a collective capacity to likely activate gene transcription at the UPRE [119]. Luman, unlike other CREB family members, localizes to the membrane and is part of a mammalian subfamily of bZIP transcription factors activated by RIP releasing the N-terminal transcription factor, allowing its subsequent nuclear translocation [reviewed in [120]]. Rat Luman expression is broad, localizing to all tissues examined, with the highest levels in liver and nervous system tissue, consistent with that reported for human Luman and supporting that it may be involved in common cellular stress responses. Nerve injury induces a higher demand for protein production and an ER stress that is associated with elevated expression of Luman in primary sensory neurons with alterations in its expression broadly corresponding to distinct injury phases where there is heightened transcriptional activity observed within the acute and prolonged regeneration phases [3,121]. These transcriptional phases were initially described as three phases—an acute injury stress response phase where Luman protein expression peaks at approximately 2 days post-lesion; a pre-regeneration phase that includes a transcriptionally quieter period around 4 days post-injury for Luman; followed by a regeneration phase, where by 7 days post-injury Luman levels peak again [121,122]. Interestingly, there is also evidence of a systemic response for Luman in uninjured sensory neurons contralateral and remote to a 7-day sciatic nerve injury. These neurons exhibit heightened Luman expression and nuclear localization and, when assayed, display an increased capacity for the elongating form of outgrowth normally observed in the regenerating neurons [121,123].

Isolating the entire coding sequence of rat Luman and demonstrating strong expression in the rat nervous system provided a necessary tool to dissect how Luman might be involved in the sensing and encoding of peripheral nerve injury in sensory neurons and whether this included regulation of the adaptive UPR/ER stress response.

### 5.1. Luman, a Retrograde Regeneration Signal

The arrival of retrograde injury signals generated at the site of the lesion help neurons switch from a homeostatic state to a regenerative state, that includes a need to generate the many proteins needed for survival and axon regeneration [89,91,124,125]. Extensive proteomics and microarray analyses identified there to be ~400 signaling networks and 39 transcription factors implicated in rat sensory axon regeneration [126]. Further, while rat sensory axon transcriptome analysis identified Luman transcripts [118], which we confirmed [3], its role as a critical regulator of regeneration-associated axon outgrowth has only recently been elucidated. Our first insights into the role of the ER-localized transcription factor Luman as a regulator of axon outgrowth in injured sensory neurons was that Luman protein localized to the ER equivalent in axons in addition to axonal Luman transcripts. In response to injury Luman can be synthesized in axons and activated with the N-terminal region (Luman[N]) being released and retrogradely transported to the cell body. This transport occurs in an importin α-dependent manner but is not linked to the proteasome. A neuronal knockdown of Luman expression using siRNA revealed that Luman regulates the regeneration-associated elongating form of axonal growth and not the highly branched growth observed in naïve neurons. Further, its expression prior to injury colocalizes with markers of the ER [3], supporting its role as an axonal ER-associated actuator of stress signals. Its location provides a mechanism whereby neurons can rapidly respond to environmental stressors such as nerve injury by activating premade transcription factors and initiating the translation of local axonal transcripts to generate additional Luman protein.

Nerve injury not only activates existing axon-localized transcription factors, but axotomy-associated calcium fluxes can initiate the translation of axon-localized transcripts coding for the retrograde transport machinery, allowing the local assembly of these modules and transport back to the nucleus [reviewed in [125]]. This axonal machinery includes constitutively expressed importin-α protein, while transcripts of another key complex component, axonal importin-β, are only translated in response to nerve injury. The association of the injury-induced axonal importin-β with importin-α creates a high-affinity NLS binding complex that Luman[N] and other activated axon-localized transcription factors can bind to, followed by retrograde transport in a dynein-dependent manner to impact transcription [127]. STAT3 has also been described to be injury-activated in the axon and transports back in a similar importin-dependent manner to mediate neuronal survival [89]. Nerve axotomy sets up a sequela of responses where Luman axonal synthesis and activation increase proximally to the site of injury, followed by association with the importin complex as part of a retrograde regeneration signal [3]. This further supports that axon autonomous injury/regeneration signals may be generated at the site of injury and appropriately relayed back to the cell body. The rapid appearance of the N-terminal portion of Luman back at the cell body and nucleus of injured sensory neurons supports its processing by RIP within nerve-associated Golgi [3]. In this manner it can serve as a rapid inductive axon injury signal and can be distinguished from the normally low levels of nuclear Luman prior to injury. That Luman is retrogradely transported as part of an importin complex via an interaction with its NLS is supported by its ability to be competed away with an excess of NLS peptide, coupled with the ability of disruptors of retrograde transport to abolish its arrival at the neuronal cell body [3].

The axonal translation of Luman transcripts in injured neurons, release of the active N-terminal region (Luman[N]) and subsequent appearance of Luman[N] in the nuclei of injured sensory neurons supports a transcriptional role in the cell body response to axonal injury [3]. But what it was transcriptionally regulating was unknown. Luman siRNA knockdown in assays of injury-conditioned versus naïve neurons however did reveal a novel injury-specific role for axonally derived Luman in the regulation of the elongating but not the branching form of axon growth, the latter normally associated with naïve neurons [3,128].

### 5.2. Luman Induces the UPR Following Axonal Injury

Luman has commonalities with components of the UPR, namely sharing similarities with XBP1s DNA binding specificities, being structurally related to ATF6 and being able to protect against UPR-related apoptosis [110]. This, coupled with injury-induced increases in neuronal Luman expression, led us to posit that it may be a regulator of the UPR associated with nerve injury [3]. Indeed, siRNA knockdown of Luman in injury-conditioned sensory neurons decreased the degree of the UPR induced in these cells and resulted in a diminished axonal/neurite outgrowth. This was in stark contrast to the lack of an affect on the cultured sensory neurons derived from naïve animals [4]. This revealed a critical role for a Luman-regulated increased UPR contributing to injury-induced axon growth/regeneration (Figure 1).

Other regulators of the UPR can also impact axon/neurite outgrowth in injury-conditioned sensory neurons. In support of this, administration of the UPR inducer tunicamycin, at levels that do not induce Luman expression in the Luman deficient (siRNA-treated) sensory neurons but at a level that induces the UPR to the level observed in axotomy-conditioned control neurons, was able to partially and significantly reverse the impaired axon/neurite growth resulting from reduced Luman expression [4].

Another regulatory factor shown to impact regeneration is cholesterol, with studies revealing positive [129] and negative [130] roles. The UPR has been linked to the regulation of cholesterol synthesis [58] through transcription factors belonging to the SREBP family [73]. The activation of SREBPs, like ATF6 and Luman, requires RIP effected by site-1 and site-2 proteases (S1P and S2P; encoded by MBTPS1 and MBTPS2) [42,108]. Decreased Luman expression by Luman selective siRNA treatment impacted the expression of genes coding for SREBP1 and S2P in a negative manner and diminished neuronal cholesterol levels [4]. The transfer of cholesterol from astrocyte to neuron has been shown to help compensate mature neurons in vivo, supporting that cholesterol can also be exogenously derived [131]. We find that exogenous cholesterol can partially compensate for the reduced cholesterol in axotomized neurons where endogenous Luman levels have been knocked down, resulting in a significantly increased ability of these cholesterol-treated neurons to extend axons/neurites in vitro. But, like tunicamycin, cholesterol supplementation did not fully compensate, suggesting additional mechanisms are involved in Luman-regulated axon regeneration. Notably, in non-targeting control siRNA-transfected axotomy-conditioned neurons, cholesterol supplementation did not induce further neurite growth, indicating that the injured-conditioned DRG neurons are able to endogenously synthesize enough cholesterol to support maximal neurite outgrowth when the UPR is not being manipulated by Luman downregulation [4].

Collectively, these findings show that we have evolved ways to sense and encode injury signals in the axonal and somal ER compartments. Luman has been identified as a major regulator of the beneficial adaptive stress response to injury associated with an elevated UPR and an increased cholesterol biosynthesis. The question remains: can we push beneficial UPR stress response further to enhance the intrinsic regenerative capacity of peripheral nerves as is seen with direct nerve stimulation strategies [6,132,133,134]?

**Figure 2:**
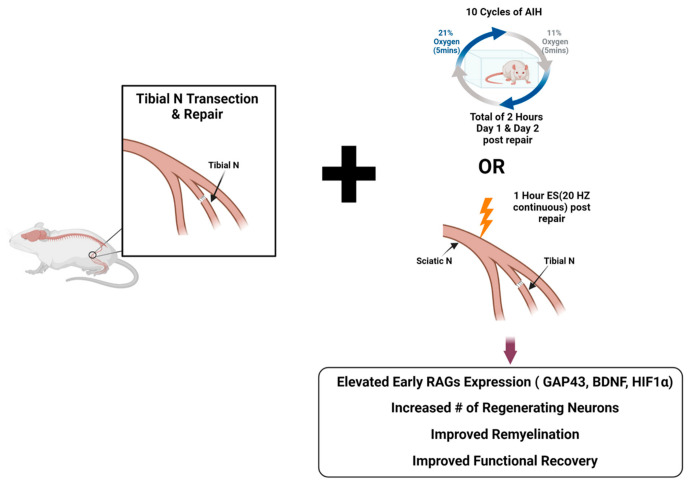
**Therapeutic AIH (tAIH) promotes nerve repair in a similar manner to that observed with direct nerve ES.** Diagram summarizing the enhanced regeneration outcomes resulting from a direct comparison of two once-daily tAIH treatments (10 cycles of 5 min of 21% O_2_ normal air alternating with 5 min of 11% low O_2_ air) on days 1 and 2 post-co-aptation repair of a transected tibial nerve versus one hour of direct nerve ES delivered just proximal to the repair site at the time of repair [5]. Both therapies delivered significant and parallel improvements in all aspects of repair examined (box below purple arrow) versus control treatments (normal 21% O_2_ air delivered for entire treatment period) or conditions (injury and repair alone). Image created using BioRender.

An emerging non-invasive therapy that induces a beneficial stress response and significantly enhances peripheral nerve regeneration in a manner akin to the current gold standard electrical nerve stimulation (ES; [6]) is therapeutic acute intermittent hypoxia (tAIH; [135], Figure 2). Evidence now supports that tAIH can induce a robust regenerative state that includes a heightened cell body response, elevated regeneration-associated gene (RAG) expression, improved remyelination and functional recovery [5]. Further, tAIH’s impact on cellular stress is evidenced by the increased expression of one of its downstream targets, hypoxia-inducible factor-1α (HIF-1α), which has been shown to play a critical role in peripheral nerve regeneration [136] and is discussed further below.

## 6. tAIH as a Promoter of Nervous System Plasticity and Repair

tAIH consists of breathing low oxygen air alternating with normal oxygen air for intermittent short periods and a brief number of cycles. It is a non-invasive therapy that was first shown to improve function in spared respiratory and non-respiratory muscle-controlling neurons in rats with partial cervical spinal cord injury (SCI) [137,138]. tAIH has strong translational potential for SCI, general organ protection and neurodegenerative disease [139,140]. It elicits sustained increases in voluntary limb muscle strength and walking distance in persons with chronic SCI [141,142,143,144]. Notably, no memory impairment is evident with tAIH [145], and it has the capacity to improve cerebral blood flow without negative impacts on cognition, cerebral perfusion pressure or oxygen carrying capacity in normal individuals [146]. It may also have the potential to improve blood pressure, doing so in individuals with obstructive sleep apnea [147]. However, we are far from understanding how broad an impact tAIH has on nervous system plasticity and functional recovery, especially given its systemic impacts [148,149].

One of the mechanisms that prompted us to directly compare it to ES is the ability of tAIH to induce long-term facilitation (LTF), a form of plasticity in rats generated by repetitive exposure to brief (5 min) episodes of mild–moderate hypoxia (11% O_2_) alternating with normoxic (20% O_2_) intervals of equal duration [150,151]. After 3–10 episodes of tAIH, LTF is manifested by an increase in phrenic motor, hypoglossal motor and sympathetic nerve amplitude that lasts for at least 2 h after AIH exposure [152,153]. This duration is within the optimal timeframe shown for electrical nerve stimulation to effect enhanced nerve repair [6,134,154]. Of note, LTF cannot be induced by continual hypoxia for the same total time as delivered in alternating episodes [155], with the acute and intermittent nature critical for inducing the LTF [156,157]. Though mechanisms rendering the benefits of AIH are incompletely understood, AIH-induced LTF requires serotonin receptor activation [158] and brain-derived neurotrophic factor (BDNF) [152,159]. In the peripheral nervous system, we recently showed that tAIH increases BDNF expression in sensory and motor neurons [5,160] and that injury-associated increases in BDNF are critical for transitioning these neurons into a robust regenerative state [161]. This also serves as an important axon Schwann cell messenger in the regulation of the myelinating capacity of Schwann cells [162]. Other key regeneration pathways are regulated by the transcription factor HIF1α, which is induced by nerve injury [136] in response to the stress induced by the lower oxygen levels associated with a peripheral nerve injury [163], with an even higher level of HIF1α expression and translocation to the nucleus observed in response to ES and AIH [5]. The heterodimeric configuration of the HIF1 transcription factor allows it to rapidly adapt to low oxygen situations by constitutively expressing its β-subunit, while oxygen levels regulate whether two prolines in its alpha subunit oxygen-dependent degradation domain are hydroxylated by HIF1α prolyl hydroxylases. This results in their subsequent ubiquitination and proteasomal degradation. Under normal oxygen levels degradation is the default pathway, while low oxygen levels inhibit the proline hydroxylase blocking the degradation of HIF1α allowing it to activate target pathways [164,165]. More recently, the conditional knockdown of HIF1α revealed a critical role in axon regeneration, with these neurons having reduced expression on one of its target genes, vascular endothelial growth factor A (VEGFA), while an exogenous application of VEGFA to the injured sciatic nerve led to increased axon regeneration [136]. Like BDNF, HIF1α also plays a role in the regulation of Schwann-mediated peripheral axon myelination and is involved in the transcription of key myelin genes such as myelin basic protein [163].

The beneficial responses to AIH occur within an optimal range of hypoxemia (9–16% inspired oxygen), coupled with a low number of episodes (3–15 episodes/day), paradigms shown to be effective in preclinical and clinical studies [166]. Although AIH holds therapeutic potential as a simple, safe therapy for many clinical disorders, its potential in preclinical models of nervous system repair is still in its infancy but bolstered by the promising and substantial outcomes in our preclinical models of peripheral nerve repair [5] and multiple sclerosis [5,167,168,169,170]. Our tAIH research employs tAIH treatment protocols within the beneficial range and are comparable to those used in the human studies [166]. One must also factor in that the duration and the diurnal cycle may impact the ventilatory plasticity induced by AIH, with shorter hypoxic episodes eliciting greater plasticity in the mid-rest phase (light), with longer episodes producing beneficial outcomes in the mid-active phase (dark) [171], highlighting that the diurnal cycle and AIH protocol must be considered when designing AIH-based therapeutic strategies.

Finally, it remains to be determined whether AIH will translate into peripheral nerve repair clinical applications as ES has [172,173,174], but the evidence supports that it has tremendous potential. The advantage that a non-invasive approach will have is the ability to easily deliver the therapy at distinct stages of regeneration, especially when the distance to regenerate is far, with the hope that it keeps the regeneration at optimal and robust levels.

Whether the beneficial adaptive stress response induced by tAIH and linked to the enhanced repair of injured axons involves Luman and its ability to regulate an adaptive UPR is currently unknown and the subject of ongoing investigations. However, gene targets of tAIH such as BDNF and HIF1α are intertwined with the UPR/ER stress responses and are both critically linked to nerve regeneration. For example, BDNF can drive the extension and branching of dendrites through a PKA-IRE1-XBP1s pathway and lead to elevated levels of somal BDNF [175], while elevated UPR/ER stress can depress BDNF expression, a response which can be mitigated with aerobic exercise [176]. BDNF has also been shown to be neuroprotective and to be essential for switching the sensory neuron from a homeostatic to a regenerative state [161,177]. The relationship between the UPR and HIF1α is more complex, with severe and prolonged hypoxia-associated HIF1α expression driving the ER stress associated with elevated CHOP and cell apoptosis contributing to the pathologies, while early or acute elevations in HIF1α expression can be protective and are consistent with what is observed in response to tAIH [5,136,178,179]. While the expression of HIF1α is normally rapidly degraded in the ERAD proteasomal system, there is also a link between chaperone-mediated autophagy (CMA) and HIF1α, whereby in fibroblasts increased CMA in response to hypoxia negatively regulated HIF1α expression. This identifies another pathway other than proteasomal [180] that regulates HIF1α degradation [181]. Finally, in other cell types, namely cardiomyocytes and human umbilical vein endothelial cells, intermittent hypoxia increases autophagy, appearing to mitigate ER stress and prevent apoptosis [182,183]. It is yet to be determined how this knowledge factors into the robust regenerative effects of tAIH on repair in the peripheral nervous system. Given these complex interrelationships, it is apparent that the design of even more effective AIH therapies for peripheral nerve repair will require continued insight into the pathways involved, so that they can be properly modulated to optimize outcomes.

## Data Availability

No unreported data presented in the review article. Statements on data availability are within cited articles from our lab.

## Abbreviations

The following abbreviations are used in this manuscript:

AIH	acute intermittent hypoxia
ATF3	activating transcription factor 3
ATF4	activating transcription factor 4
ATF6	activating transcription factor 6
ATP	adenosine triphosphate
BDNF	brain-derived neurotrophic factor (BDNF
bZIP	basic leucine zipper
CARE	CCAAT-enhancer binding protein-activating transcription factor response elements
CHOP	CCAAT-enhancer-binding protein homologous protein
CREB	c-AMP response element binding protein
JIP	c-Jun amino-terminal kinase interacting protein
JNK	c-Jun amino-terminal kinase
cAMP	cyclic adenosine monophosphate
COPII	coat protein II
CRE	cyclic AMP response element
DLK	dual leucine zipper kinase
DRG	dorsal root ganglion
EDEM	ER degradation-enhancing alpha-mannosidase
ERAD	ER-associated degradation
ERSE	ER stress element
ER	endoplasmic reticulum
ES	electrical nerve stimulation
eIF	eukaryotic translation initiation factor
eIF2	eukaryotic translation initiation factor 2
GADD34	DNA damage-inducible protein 34
GRP78	glucose-regulated protein 78
HERP	homocysteine-induced endoplasmic reticulum protein
HIF1α	hypoxia-inducible factor-1α
HSV	herpes simplex virus
IRE1	inositol-requiring protein-1
INSIG	insulin-induced gene
LDL	low-density lipoprotein
LTF	long-term facilitation
NLS	nuclear localization signal
PKA	protein kinase A
PNS	peripheral nervous system
PCSK9	proprotein convertase subtilisin kexin type 9
PERK	protein kinase RNA-like ER kinase
PP1	type I protein phosphatase
RAG	regeneration-associated gene
RIP	regulated intramembrane proteolysis
RNase	endoribonuclease
S1P	site-1 protease
S2P	site-2 protease
SRP54	signal recognition particle 54
SREBs	sterol regulatory element-binding proteins
STAT3	signal transducer and activator of transcription 3
SCI	spinal cord injury
tAIH	therapeutic acute intermittent hypoxia
UPR	unfolded protein response
UPRE	UPR element
VEGFA	vascular endothelial growth factor A
XBP1	X-box binding protein 1
XBP1s	spliced form of XBP1
